# Draft Genome Sequence of *Pacificimonas aurantium* Type Strain JLT2012, Isolated from the Seawater of the Pacific Ocean

**DOI:** 10.1128/genomeA.00755-17

**Published:** 2017-08-24

**Authors:** Guangrong Tang, Rong Cui, Yu Tian, Xiaopei Lin

**Affiliations:** a China National Offshore Oil Corporation Co, Ltd., Zhanjiang Branch, Zhanjiang, China; b State Key Laboratory of Marine Environmental Science, Institute of Marine Microbes and Ecospheres, Xiamen University, Xiamen, China

## Abstract

Type strain JLT2012 was isolated from the southeastern Pacific. Here, we report the draft genome sequence and the initial findings from a preliminary analysis of strain JLT2012, which represents a novel species and should be classified in the existing genus *Pacificimonas*.

## GENOME ANNOUNCEMENT

Type strain JLT2012 (LMG 27361^T^ = CGMCC 1.12399J^T^), which was isolated from surface waters (depth, 50 m) in the southeastern Pacific, is a rod-shaped yellow-pigmented Gram-negative bacterium displaying gliding motility (1). Phylogenetic analysis using 16S rRNA gene sequences showed that strain JLT2012 formed a distinct lineage within the genus *Pacificimonas* (formerly known as *Pacificamonas*) and shared the highest sequence similarity with the type strain *Pacificimonas flava* JLT2015 (96.0%). Members of the *Sphingomonadaceae* are characterized by a smaller cell size than that of copiotrophic bacteria and display an oligotrophic strategy with low rates of growth in the nutrient-limited marine environment (2). Here, we report the draft genome sequence of this strain.

The genome sequence of JLT2012 was determined via the Illumina HiSeq 2500 system. The paired-end reads generated by the Illumina sequencer were assembled by using Velvet version 1.2.03 (3). Coding sequences (CDSs) were predicted using Prodigal version 2.60 (4). The draft genome of strain JLT2012 contained 3,352,426 bp distributed in 7 DNA scaffolds, with a GC content of 64.15%. The scaffold *N*_50_ was 3,041,156 bp, the longest scaffold was 3,041,156 bp, and the shortest scaffold was 835 bp. Putative functions from functional Clusters of Orthologous Groups (COG) were assigned to 82.1% of the candidate genes. Rapid Annotation using Subsystems Technology (RAST) (5) was used to perform annotation of the assembly data. The genome consisted of one linear chromosome with 3,198 protein-coding genes (CDSs), 46 tRNA genes, and 3 rRNA operons. We predicted the tRNA genes and the rRNA genes by tRNAscan SE version 1.31 (6) and RNAmmer version 1.2 (7), respectively.

Genes putatively encoding a choline dehydrogenase and related flavoproteins were present in the genome. A portion of genes involved in the biodegradation of xenobiotics (about 5.1% of the whole genome) were also located in the JTL2012 genome from the results of KEGG pathway classification, and the xenobiotic agents include nitrotoluene, atrazine, aminobenzoate, styrene, caprolactam, fluorobenzoate, and dioxin. Genes for dicarboxylate and tricarboxylate transporters, nitrate reductase, and sulfate reductase were also present in the genome of strain JLT2012.

Comparison with genome sequences available in RAST showed that *Sphingobium japonicum* UT26S (score, 505) and *Sphingomonas wittichii* RW1 (score, 439) were the closest neighbors of strain JLT2012.

### Accession number(s).

The type strain JLT2012 shotgun genome sequence has been deposited at GenBank under the accession no. NFZT00000000. The version described in this paper is the first version, NFZT01000000.
